# Proteomes and Signalling Pathways of Antler Stem Cells

**DOI:** 10.1371/journal.pone.0030026

**Published:** 2012-01-18

**Authors:** Chunyi Li, Anne Harper, Jonathan Puddick, Wenying Wang, Chris McMahon

**Affiliations:** 1 Developmental Biology Group, AgResearch Ltd, Invermay Agricultural Centre, Mosgiel, New Zealand; 2 State Key Laboratory for Molecular Biology of Special Economic Animals, Changchun, China; 3 Waikato Mass Spectrometry Facility, University of Waikato, Hamilton, New Zealand; 4 Developmental Biology Group, AgResearch Ltd, Ruakura Agricultural Centre, Hamilton, New Zealand; University of Massachusetts Medical School, United States of America

## Abstract

As the only known example of complete organ regeneration in mammals, deer antler in the growing or velvet phase is of major interest in developmental biology. This regeneration event initiates from self-renewing antler stem cells that exhibit pluripotency. At present, it remains unclear how the activation and quiescence of antler stem cells are regulated. Therefore, in the present study proteins that were differentially expressed between the antler stem cells and somatic cells (facial periosteum) were identified by a gel-based proteomic technique, and analysed using Ingenuity Pathway Analysis software. Several molecular pathways (PI3K/Akt, ERK/MAPK, p38 MAPK, etc.) were found to be activated during proliferation. Also expressed were the transcription factors POU5F1, SOX2, NANOG and MYC, which are key markers of embryonic stem cells. Expression of these proteins was confirmed in both cultured cells and fresh tissues by Western blot analysis. Therefore, the molecular pathways and transcription factors identified in the current study are common to embryonic and adult stem cells. However, expression of embryonic stem cell transcription factors would suggest that antler stem cells are, potentially, an intermediary stem cell type between embryonic and the more specialized tissue-specific stem cells like those residing in muscle, fat or from a hematopoietic origin. The retention of this embryonic, pluripotent lineage may be of fundamental importance for the subsequent regenerative capacity of antlers.

## Introduction

The annual full regeneration of deer antlers is unique among mammals and the evidence to date indicates that it is a stem cell based process [1], [2], [3]. Antler regeneration occurs in yearly cycles consisting of growth, calcification, antler skin (also known as velvet) shedding and antler casting [4]. During the growth phase, antlers are made up of cartilage and bone infiltrated with blood vessels and nerve networks and covered by a velvet skin [5]. Generally, stem cells play a crucial role in tissue and organ formation [6] and in regeneration [7]. Deer antler provides a single organ model in which growth and development are controlled by the proliferation and differentiation of tissue specific stem cells with embryonic like properties [8], [9]. Antler stem cells are an invaluable model for investigating these fundamental biological phenomena. A recent study [10] showed that the pool of stem cells from which antler regeneration initiates resides in the periosteum of a permanent bony extension from the deer skull termed the pedicle (Figure S1A). Pedicles that are deprived of the enveloping periosteum do not regenerate antlers (Figure S1B). Thus the cells are termed the pedicle periosteum cells (PP cells). The antler bud forms from the pedicle periosteum and the velvet antler then grows from the cells of the mesenchyme located at the tip of the main beam and the tines once the antler branches form [11]. The exact molecular mechanism by which velvet antler develops from the pedicle is not yet fully understood. Growth of the pedicle itself is initiated during puberty from a different pool of stem cells located in a zone named the antlerogenic periosteum (AP cells; Figure S1C), which covers a crest in the deer skull located just above the eye socket [12]. Removal of the AP prior to pedicle initiation stops pedicle and antler growth, while transplantation of the AP induces ectopic pedicle and antler formation (Figure S1D; 10–12).

Once the pedicles reach approximately 6 cm in height in red deer, the first antlers emerge from their apices [13], [14]. Subsequent antler growth cycles are under the control of androgen hormones [15], [16] and influenced by environmental conditions [17], [18]. In utero, a primordial pedicle begins to grow at about 60 days of gestation and continues to develop until about 100 days when growth slows. By the time the calf is born the pedicles are not noticeable [19]. There is evidence to suggest that the AP in the adult is a piece of retained embryonic tissue, which may, therefore, retain embryonic stem cell capabilities, i.e. pluripotency [8]. In support, we have stimulated differentiation of AP cells and PP cells into chondrocytes, adipocytes, osteoblasts and possible neural cells in vitro [9]. At present, it remains unclear how the AP and PP stem cells are regulated. Therefore, we sought to identify candidate proteins in AP cells and PP cells that regulate the activation and quiescence of antler stem cells in the current study. Expression was then compared against facial periosteum cells (FP cells) derived from the nasal bone of the deer head as the reference tissue.

## Methods

### Ethics Statement

All studies were approved by the Invermay Animal Ethics Committee (Agresearch Ltd) in application 482.

### Tissue sampling

Antlerogenic periosteum (AP), PP and FP were collected from red deer heads immediately after slaughtering in May (early winter in the southern hemisphere) and October (late spring), according to the protocol described by Li and Suttie [20]. Briefly, to collect the AP from a yearling male, a crescent-shaped incision was made on the scalp skin 2 cm medial to the frontal crest, skin was separated from the frontal bone and reflected laterally to expose the AP. The AP was then peeled off from the underlying bone following incisions cut on the periosteum (Figure S1C).

To collect the PP from a 2-year-old male, a crescent-shaped incision was made on the deer scalp skin 2 cm medial to the base of a pedicle, and a second skin incision was made surround the pedicle shaft 2 cm distal to the pedicle tip. The third skin incision positioned on the skin of the pedicle medial surface, and started from the second incision and terminated when it met the first one at the base of the pedicle. To expose pedicle bone, the enveloping skin of the pedicle was separated from the bony core through these incisions and reflected laterally. The PP was then divided into strips of approximately 0.5 cm in width along the longitudinal axis of the pedicle, and each strip was then peeled off (Figure S1A).

To collect the FP from a yearling male, a 4–6 cm long skin incision was made parallel to the midline of the nose on one side of the face. This incision was then continued medially from the both ends until meeting at the midline of the nose. A flap of skin was separated and reflected medially to expose one side of the nasal bone. The facial periosteum was then cut into small strips (0.5×3 cm) and peeled off from the underlying bone.

### Primary culture of AP cells, PP cells and FP cells

All reagents were purchased from Invitrogen for primary culture of the periosteal cells. The culture was carried out as per Li et al [21]. Briefly, each type of periosteum was minced using two scalpels, digested in a digestion medium (25 ml DMEM medium containing 2.5% FBS, 200 units/ml collagenase, 100 U/ml penicillin and 100 g/ml streptomycin) and cultured in a culture medium (10 ml DMEM medium containing 10% FBS, 100 U/ml penicillin and 100 g/ml streptomycin). Cells were trypsinized when confluent and transferred into T75 culture flasks for two days at the density of 2×10^4^ cells/ml before being frozen down. Cells were stored in liquid nitrogen in the frozen medium (DMEM containing 15% FBS, 5% DMSO, 5% glycerol).

No effort was made to further purify sub-cell populations from each tissue type as it is currently unknown whether antler generation or regeneration is derived from a single or all cell populations of periosteum tissue. So far a mixture of all the cell populations from each periosteum tissue sample has been termed AP cells, PP cells or FP cells [2]. Cells were retrieved from storage and grown in the culture medium to sub-confluence in T75 flasks before use.

### Two dimensional gel electrophoresis

All chemicals were obtained from Sigma unless stated otherwise. For each cell type up to 1.7×10^6^ cells were harvested by treating with trypsin for 2 minutes followed by re-suspension in 10 ml sterile PBS containing protease inhibitor cocktail (Roche). Cells transferred to 50 ml tubes (Becton & Dickinson) were spun at 400× *g* for 5 min and washed in 10 ml of the PBS solution three times. Finally, cells were re-suspended in 100 µl of PBS with protease inhibitors, transferred to 1.5 ml microfuge tubes (Axigen) and frozen at −80°C overnight. Cells were lysed by thawing on ice and then sonicating twice for 10 seconds. Cell debris was removed by centrifugation at 4°C and 1600× *g* for 10 min. The supernatant was transferred to a fresh microfuge tube. Proteins were precipitated by adding 500 µl of acetone with 10% trichloroacetic acid and 20 mM dithiothretol to the cell lysate and storing at −20°C overnight. The proteins were harvested by centrifugation at 4°C and 16× *g* for 15 min. The supernatant was removed without disrupting the pellet and discarded. The pellet was washed three times by resuspension in 600 µl cold acetone and centrifuging as above. The pellet was left to air dry for 1 h.

The proteins in each pellet were solubilised by adding 50 µl of PBS with protease inhibitors as above and vortexing, followed by 450 µl of DeStreak solution (GE Healthcare) and vortexing. Protein concentration was measured using EZQ Protein Quantitation Kit (Invitrogen Molecular Probes).

For the IEF phase 100 µl of a 20 mg/ml protein solution containing 6 µl of IPG buffer pI 4 to 7 range or pI 3–10 range, was cup-loaded onto a 18 cm pI 4 to 7 range or pI 3–10 range, IPG strip (GE Healthcare). Samples were electrophoresed at 300 V, 600 V, 800 V, 1000 V and 2000 V for 1 h at each voltage and then 3500 V for 18 h. For the second phase IPG strips were prepared as per the manufacturer's instructions and placed on a 24 cm XL 12–14% gradient gel (GE Healthcare) and run as per the manufacturer's instructions. Ten µl each of rainbow molecular weight markers (GE Healthcare) were run at either side of the IPG strip. Gels were fixed for 1 h in 10% acetic acid, 40% ethanol, stained overnight in colloidal coomassie blue containing 17% ammonium sulphate, 34% methanol, 2% phosphoric and 0.1% brilliant blue G250 acid, and destained for 1 to 2 h in 7% methanol.

Gels were first run over a broad pI range of 3–10 and then pI 4–7 range when it was found that most of the antler stem cell proteins were in that region of the gel. The pI 4–7 range gels were used for analysis and subsequent protein spot removal for identification. To control for variation between gels three gels were run per cell type and used to make a matchset in PDQUEST (Bio Rad) as per the manufacturer's instructions. The matchsets were then used for the comparison of the proteomes of the three cell types. Using PDQUEST v 6.2.1, protein spots were identified as being either present in AP cells or PP cells but not FP cells, or at least two fold overexpressed when compared with FP cells. Gel samples were excised from the gel using a fresh 1 ml pipette tip and analysed by Mass Spectrometry at the Waikato University MS facility. Only those proteins which were over-expressed were selected and sent for identification.

### In-gel Tryptic Digest

Gel pieces were destained in 1∶1 25 mM NH_4_HCO_3_/acetonitrile (ACN) before being dehydrated in ACN and air dried. The gel pieces were rehydrated at 4°C in 5 ng/µl Trypsin Gold (Promega) resuspended in 25 mM NH_4_HCO_3_. After removal of excess trypsin solution, gel pieces were incubated at 37°C for 6 h. The resulting peptides were extracted overnight at 4°C in 20% ACN/0.1% TFA.

### Peptide Sample Preparation

Peptides were prepared for Matrix Assisted Laser Desorption Ionisation-Time of Flight (MALDI-TOF) mass spectrometry by applying 0.5 µl onto a thin layer of α-cyano-4-hydroxy-cinnamic acid on a 600 µm Anchorchip™ (Bruker Daltonics) target. The dried sample was then washed by applying 0.1% TFA in 10 mM NH_4_H_2_PO_4_ for around 5 seconds before removing.

### Peptide Mass Fingerprinting

Peptide masses were determined on a Bruker Daltonics AutoFlex II™ MALDI-TOF mass spectrometer operated using FlexControl™ (Bruker Daltonics). The instrument was calibrated against a peptide calibration standard (Bruker Daltonics) which was loaded in the same manner as the samples, onto calibanchors, located at the centre of every four sample anchors. Sample spectra were acquired over a 500 to 4,000 m/z range by summing 500 shots, with an acceleration voltage of 19 kV and a reflector voltage of 20 kV. Pulsed ion extraction of 80 ns was used to build up the concentration of ions in the ion source and ions below 500 m/z were suppressed to avoid detector saturation from matrix ions. Spectra were automatically annotated by FlexAnalysis™ (Bruker Daltonics) to pick the mono-isotopic peaks within 800 and 4,000 m/z, and Mascot™ database searches were performed via BioTools™ 3.0 (Bruker Daltonics), searching the NCBI nr database.

### Analysis of identified proteins

Ingenuity Pathway Analysis (IPA) software (www.ingenuity.com) was used to determine the relationships between the identified proteins. As the software can only be applied to human, mouse, rat or dog proteins, an orthologue from one of these species was obtained using a BLAST search of the NCBI database. The data from the two proteomes were imported separately into the software package and the analysis carried out as per the manufacturer's recommendations. IPA software produces network diagrams with both the proteins in the data set and proteins which could be part of the network as determined by the analysis. IPA uses a right tailed Fishers Exact Test to determine the probability of the proteins in a data set having a functional relationship. Probabilities (p value) of less than 0.05 were considered significant. IPA also calculates a ratio which indicates the strength of association with a canonical pathway. From these two numbers IPA determines the most significant canonical pathways associated with the dataset.

### Western blots

Western blots were performed to detect S100A4, LGALS1, NANOG, SOX2, POU5F1 (OCT4), MYC and MYCN in the cultured cells and in the tissues harvested from AP, PP and FP. We also probed for VEGFA as it has been observed in the growing tip of antler [22], [23]. For the cell-derived protein samples 10 µl of the samples were used for the 2D gels. For the tissues, samples were harvested as described above and immediately frozen in liquid nitrogen and then homogenized while frozen using a SPEX 6750 freezer mill (Wolf Laboratories Limited, UK). Fifty mg of this tissue powder was suspended in 1 ml of extraction buffer of 8 M urea, 4% CHAPS, 40 mM Tris base, 20 mM DTT and protease inhibitor cocktail (Roche). The mixture was then sonicated on ice 4 times for 30 second periods with a rest time of 1 min on ice to cool. Insoluble material was removed by centrifugation at 4°C and 1600× *g* for 10 min. The supernatant was transferred to a fresh microfuge tube and precipitated as for the cell derived proteins. The precipitated proteins were made up to a concentration of 20 mg/ml in the IEF buffer as above. Concentration was checked using the EZQ Protein Quantitation Kit (Invitrogen Molecular Probes). 10 µl of this was loaded onto the gel for each sample.

NuPage Bis-Tris 4–12% gradient gels with MES buffer (Invitrogen) were used to separate the proteins and the Xblot Mini cell system (Invitrogen) was used to perform the transfer to nitrocellulose membrane following the manufacturer's protocols. The antibodies used were rabbit anti S100A4 (Dako), mouse anti LGALS1 and VEGFA (Sigma), rabbit anti MYC and MYCN (Santa Cruz), and goat anti POU5F1, SOX2 and NANOG (Santa Cruz). Secondary antibodies were all from Sigma conjugated with alkaline phosphatase. Primary antibodies were diluted 1 in 5000, secondary antibodies 1 in 10,000. Bands were detected using the Western Breeze Chromogenic kit (Invitrogen).

Western blots were also performed using the cell culture media following the protocol above after removing albumin and IgG using a commercial kit (GE Healthcare code RPN6300). The samples were then concentrated in a Vivaspin 500 MWCO 3000 (GE Healthcare) concentrator as per the manufacturer's protocol to a concentration of 20 mg/ml determined by the EZQ Protein Quantitation Kit. Ten µl was loaded onto the gel for each sample.

## Results

### Two dimensional gel electrophoresis

**Figure 1:**
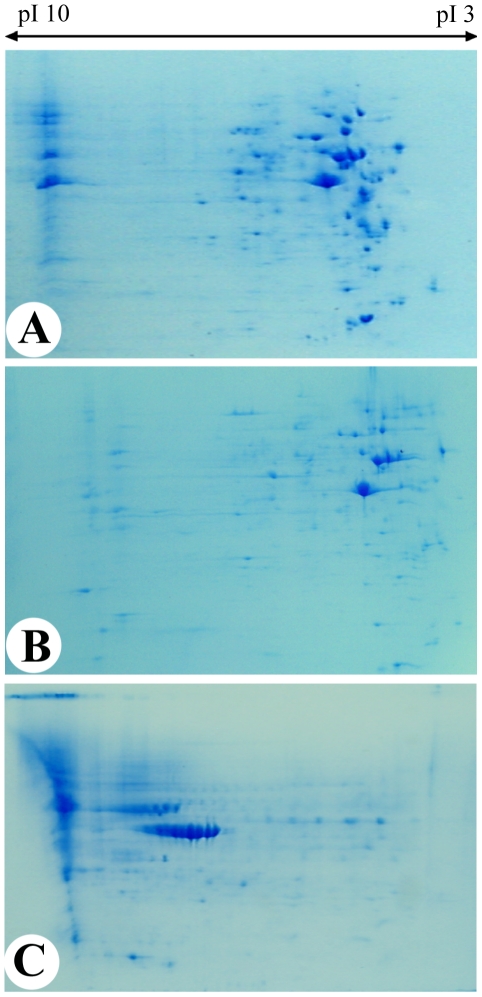
Two dimensional electrophoresis gels. The gels are over the broad pI range of 3 to 10 for AP cells (1A), PP cells (1B) and FP cells (1C). Note that most of the proteins in AP cells and PP cells were in the low pI region of the gel. AP cells, antlerogenic periosteum cells; PP cells, pedicle periosteum cells; and FP cells, facial periosteum cells.

**Figure 2:**
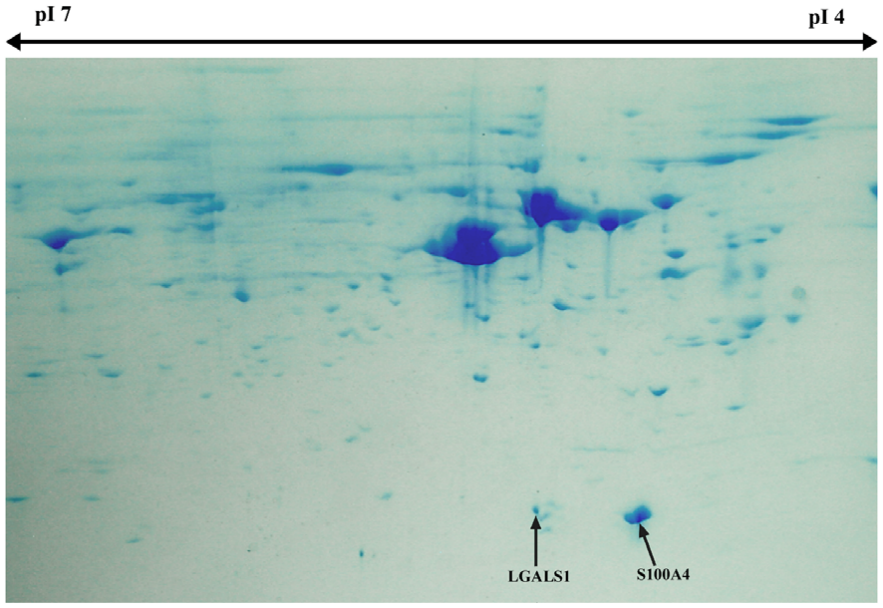
Two dimensional electrophoresis gel for AP cells. The gel highlights the strong expression of S100A4 and LGALS1 proteins in AP cells. AP cells, antlerogenic periosteum cells.

An initial analysis of the proteome of the cells over a broad pI range of 3 to 10 units indicated that the large majority of the proteins from AP cells and PP cells had a pI below 7 (Figure 1A and 1B). The FPC proteome was distributed mainly across the 7 to 10 range (Figure 1C). Further analysis was carried out over the pI range 4 to 7 to maximize resolution (Figure 2).

Sixty six proteins were identified from the AP cells and 98 proteins from the PP cells. The proteins expressed by the AP cells and PP cells were listed in Tables S1 and S2 respectively, including the number of isoforms and the fold change compared with FP cells. Tables S1 and S2 also provide a key for the protein names used in this manuscript.

Proteins that mediate signal transduction and were expressed in AP cells were S100A4 (Figure 2) which was not present in PP cells or FP cells, and LGALS1 which was 15-fold overexpressed compared with FP cells. SPARC was only expressed in AP cells. ANXA4 and ANXA5 were 15-fold and twofold upregulated in AP cells respectively. Proteins identified in PP cells included LGALS1, which was overexpressed by 20-fold in PP cells, GSN, which was overexpressed by 10-fold compared with FP cells and IL8, which was expressed in PP cells only. ANXA4 and ANXA5 were each two-fold overexpressed in PP cells compared with FP cells. ANXA1 was upregulated two-fold and ANXA2 was expressed in PP cells, but was absent in both AP cells and FP cells; and COL6A1 was upregulated 10-fold in PP cells compared with FP cells. Finally, COL1A1 was expressed in both AP cells and PP cells, but not in FP cells.

### Ingenuity Pathway Analysis (IPA)

For AP cells, the significant signalling pathways were PI3K/Akt signalling (p<0.001, ratio = 0.038), 14-3-3 signalling (p = 0.002, ratio = 0.0.32), regulation of actin based motility by Rho (p = 0.015, ratio = 0.033) and ERK/MAPK signalling (p = 0.02, ratio = 0.022).

For PP cells, the significant signalling pathways were actin cytoskeleton signalling (p<0.001, ratio 0.027), ERK/MAPK signalling (p<0.001, ratio = 0.027), p38 MAPK signalling (p = 0.002, ratio = 0.032) and PI3K/Akt signalling (p = 0.004, 0.023).

**Figure 3:**
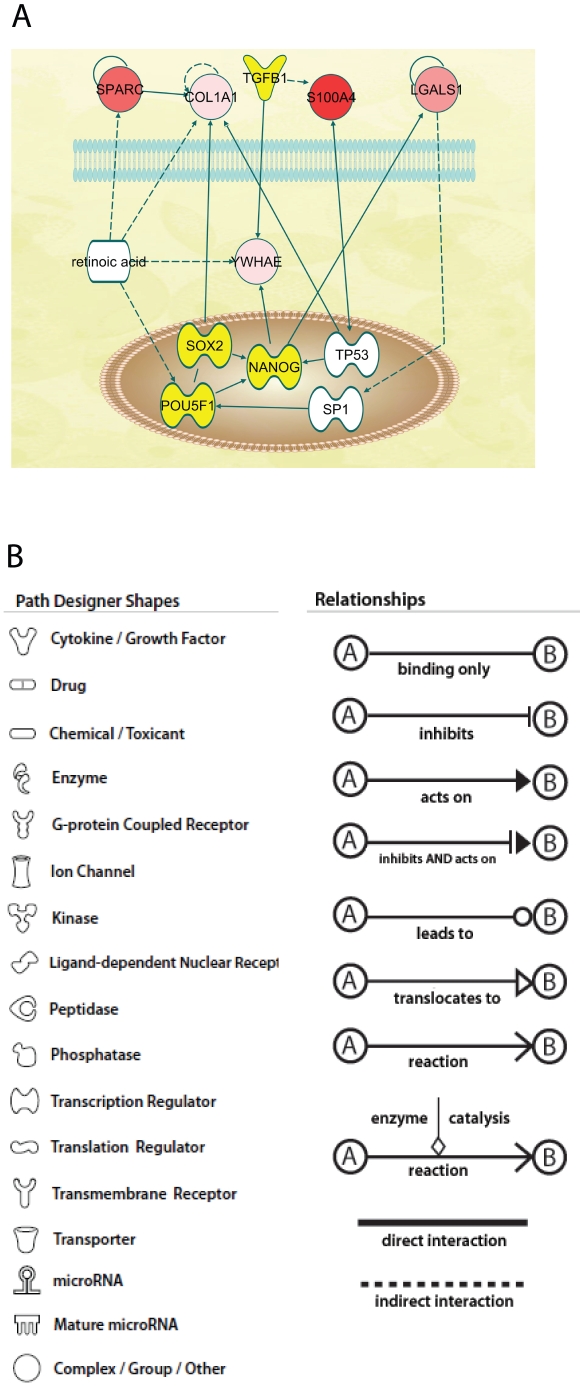
Network diagram for AP cells produced by IPA analysis. **A.** The diagram shows the potential interactions of the extracellular proteins with NANOG, SOX2 and POU5F1. Proteins detected in the proteomic study are shown in grey to red. The deeper the red the greater the level of expression. Proteins indicated by IPA analysis and subsequently detected by western blots are shown in yellow. Proteins shown in white are indicated as involved in the network but have not been detected yet. AP cells, antlerogenic periosteum cells. **B.** Key from IPA (www.ingenuity.com) to network diagram molecules and relationships between them in this Figure and in Figures 4, 5, and 6.

**Figure 4:**
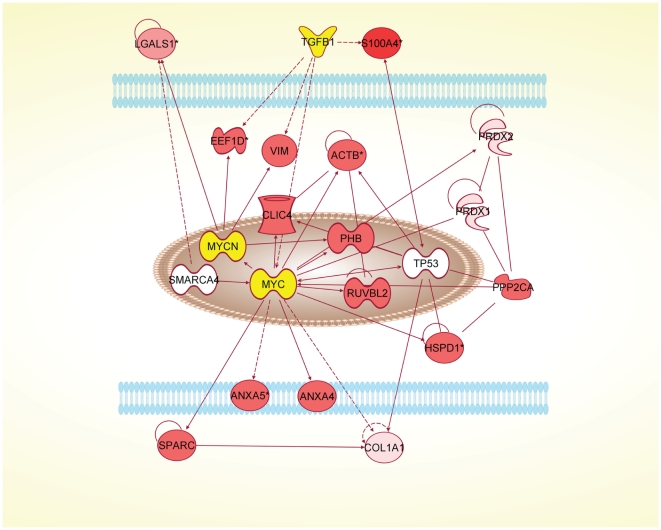
Network diagram for AP cells produced by IPA analysis. The diagram shows the potential interactions of the extracellular proteins with MYC and related transcription factors. AP cells, antlerogenic periosteum cells.

**Figure 5:**
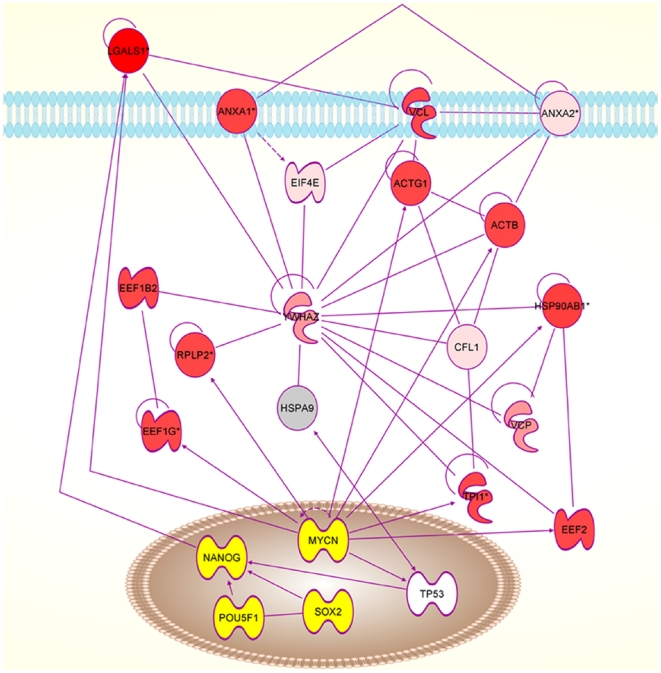
Network diagram for PP cells produced by IPA analysis. The diagram shows the possible interactions of LGALS1 with NANOG and related transcription factors, either directly or through 14-3-3 signalling (YWHAZ). PP cells, pedicle periosteum cells.

**Figure 6:**
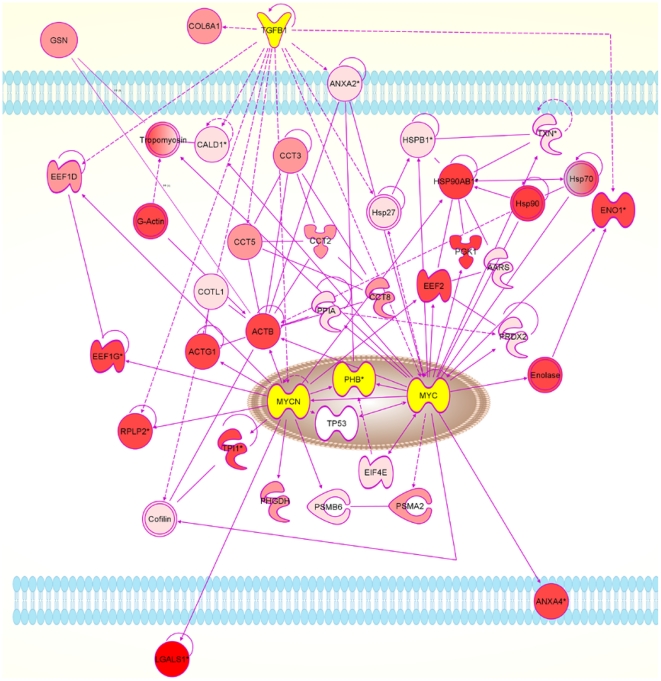
Network diagram for PP cells produced by IPA analysis. The diagram shows the possible interactions of LGALS1 with MYC and related transcription factors. PP cells, pedicle periosteum cells.

Proteins identified subsequently by Western blot were not included in the datasets used to calculate p-values and ratios because no expression value could be assigned from the qualitative data. One large network could be constructed for each antler stem cell type: AP cells (Figure S2) and PP cells (Figure S3). From these, the possible interactions of the extracellular proteins and transcription factors could be determined (Figure 3, 4, 5 and 6).

In AP cells, IPA indicated that retinoic acid (RA) could be interacting with SPARC, COLA1, POU5F1, and YWHAE (Figure 3). In the PP cells proteome, several RA associated proteins were found (CRAPB2, CCT5, EEF1G, EEF1D, LAP3 and KRT1).

### Western blotting

**Figure 7:**
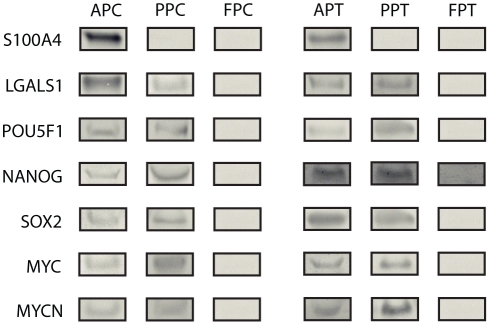
Western blot gels for AP, PP and FP cells and tissues. The gels show the signalling molecules and transcription factors detected in this study. Note that the results from the cultured cells and from the tissues were consistent: S100A4 only expressed in AP; LGALS1, POU5F1, Nanog, SOX2, MYC, and MYCN expressed only in AP and PP, but not in FP. AP, antlerogenic periosteum; PP, pedicle periosteum; and FP, facial periosteum.

The network diagrams produced by IPA analyses indicated the involvement of the transcription factors MYC, MYCN, POU5F1, SOX2 and NANOG, which are already known for their roles in stem cell biology. Therefore, we performed Western blots and confirmed their presence in both AP and PP cells and tissues (Figure 7).

**Figure 8:**
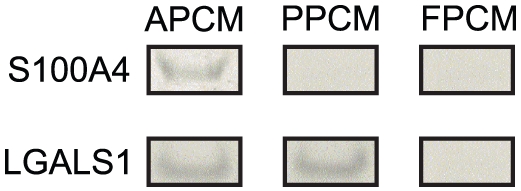
Western blot gels for AP, PP and FP cell culture medium (CM). The results using cultured medium not only further confirmed the results using cells and tissues (Figure 7, only expressed in AP and PP, but not in FP), but also showed that S100A4 and LGALS1 were the secreted molecules. AP, antlerogenic periosteum; PP, pedicle periosteum; and FP, facial periosteum.

The Western blots also confirmed expression of the following factors: S100A4 and LGALS1 in the AP cells and tissues and LGALS1 in the PP cells and tissues (Figure 7). LGALS1 was also detected in the culture media of both AP and PP cells but not FP cells; and S100A4 only in the medium of AP cells (Figure 8), suggesting that these proteins are secreted by the antler stem cells in vitro. VEGFA was not detected in any of the tissues (data not shown).

## Discussion

This is the first comprehensive study of the proteins up-regulated by antler stem cells when cultured in vitro. The results show some similarities with work published to date which concentrated on the cells in the growing tip of later stage velvet antler [24]. The present study gives us an insight into the early events of antler regeneration when the cells of the PP are self-renewing in readiness to form the antler bud, and the even earlier event when the pedicle is beginning to form from the AP [25], [26], [27]. We are aware that isolation and culture of these cells in vitro, which altered the stem cell niche, could have influenced protein expression. However, the similarities between our results and those of other workers in field studying whole antler tissue is reassuring even though most previous work focuses on later stages of antler growth. Confirmation of specific proteins identified using Western blotting in the tissues and in the cell culture media further supports our 2DE results.

S100A4 was strongly expressed by AP cells and not by PP cells or FP cells. It was also found in AP tissue and APC media only, suggesting a signalling role in AP cells. This molecule has intra and extracellular activity; exists in homodimeric and oligomeric forms, each with different activities. S100A4 is regarded as a mediator of metastasis and studies on patients have shown a correlation between S100A4 upregulation and poor prognosis in cancer [28], [29]. This molecule modulates cell motility, effecting cell polarization through the regulation of myosin-IIA filament assembly [30]. It also interacts with liprin 1 [31] affecting cell motility, p53 [32] affecting cell survival and annexin II [33], [34] causing remodelling of the extracellular matrix (ECM) and inducing angiogenesis. In mouse embryogenesis S100A4 is involved in the differentiation of mesenchymal tissues and the development of bone and foetal macrophages [35]. It binds to myosin light chain and F actin [36], which we find are upregulated by AP cells. Therefore, S100A4 could be involved in cell motility when APC cells migrate to form the pedicle. S100A4 also upregulates TP53 and, therefore, could be mediating apoptosis, which has been found to be important in antler regeneration [37]. At this stage we cannot determine whether S100A4 over-expression is involved in cell motility or apoptosis or both in the case of antler development.

LGALS1 was found to be 15-fold over-expressed in AP cells and 20-fold overexpressed in PP cells. It was also found in the media of both cell types and not in FPC media. This suggests an important role for LGALS1 as a signalling molecule in the antler stem cells. LGALS1 is a carbohydrate binding protein with a variety of structure dependant functions in the cell and the extracellular space [38]. Its expression is regulated by various effectors including retinoic acid (RA, [39]). LGALS1 modulates the immune response [40], [41] and may contribute to immune privilege in tumours. In tumours, LGALS1 is regarded as a predictor of metastasis [38] and a treatment target. LGALS1 regulates myotube growth in regenerating skeletal muscle [42]. In the dimeric and monomeric form it promotes the growth of various nerve tissues [43]. The molecule was also identified by Park et al in the antler tip [44]. LGALS1 stimulates myoblast differentiation of AP cells in vitro (our unpublished results). Our IPA analyses point to interactions of LGALS1 with NANOG, MYCN and SMAD4 via the 14-3-3 signalling pathway in AP cells. Similarly, the IPA analyses indicate that LGALS1 interacts with NANOG and MYC in PP cells. The presence of LGALS in both APC and PPC cell types suggests that this protein is either regulating or regulated by MYC, MYCN and/or NANOG, but it remains unclear how this regulating influences activation of the antler and pedicle stem cells.

SPARC is another extracellular protein expressed only in AP cells. SPARC is a multifunctional protein with roles in skeletal development and a cell type dependant effect on proliferation. SPARC consists of three modules [45] each of which can be cleaved and the resulting peptides remain active with distinct functions. SPARC upregulates COL1A1, which we also found expressed in AP cells, but not in FP cells. TGFB1 stimulates the expression of SPARC [46] and also causes differentiation of AP cells to osteoblasts [47]. Our results suggest that SPARC is upregulated by RA and/or TGFB1 [48] and this leads to the expression of COL1A1. COL1A1 is an extracellular matrix protein found in most connective tissues particularly bone, cornea, dermis and tendon. It is also found in growing antler tip [49]. SPARC can also be down-regulated by MYC [50], [51] which would inhibit expression of COL1A1. SOX2 also downregulates COL1A1 [52]. Thus the transcription factors promote the proliferation of the AP cells without differentiation into osteoblasts until an as yet unknown signal changes the relative expression levels of the proteins.

The identification of IL8 in PP cells but not in FP cells or AP cells is interesting as IL8 is an inflammatory cytokine [53], is angiogenic [54] and tumourigenic [54], [55]. IL8 has been shown to mobilize hematopoietic stem cells [56]. Our analyses indicate that IL8 could upregulate MYC via the PI3K/Akt pathway and is, therefore, involved in cell cycle progression and thus proliferation of the PP cells. Given that a limited number of PP cells (around 3.3 million cells) can generate 10 kg or so antler tissue mass within 60 days [9], novel mitogenic factors would be indispensible.

Another extracellular protein of interest in PP cells is COL6A1 (overexpressed 10-fold) which is upregulated by TGFB1 via SMAD3 [57] suggesting a role in ECM remodelling but the relevance of this to antler regeneration is unknown as yet. PI3K/Akt and ERK/MAPK signalling appears common to both AP cells and PP cells as does some form of signalling involving the actin cytoskeleton. 14-3-3 signalling is active in AP cells and ERK/MAPK in PP cells.

PP cells overexpress the extracellular protein GSN (10-fold overexpressed) which upregulates cell motility. GSN binds, cleaves and caps the barbed end of actin filaments in a calcium dependant manner [58]. GSN binds both TPM2/3 and CALD1, which are upregulated in the PP cells. Also involved in cell motility is the nuclear protein CFL1 [58] expressed only by PP cells. In PP cells the upregulation of actin cytoskeleton signalling components GSN, CFL1, CALD1, TPM2 and TPM3 indicates a high degree of motility compared with the FP cells and fits with the notion that the PP cells migrate to form the mesenchymal layer during the development of the early antler bud and provide a pool of progenitor cells for subsequent antler growth.

A group of proteins which are over-expressed between two and fifteen fold by antler stem cells are the annexins. The functions of annexins (ANXAs) are not yet clearly defined although differential expression of ANXAs is often observed in various disease states. ANXA1 is associated with the inflammatory process [59], has anti-proliferative effects activated by various phosphorylation dependant pathways and can mediate apoptosis via the dephosphorylation of BAD [60]. Molnar et al [49] found strong expression of ANXA2 in the growing antler tip. Park et al [24] detected expression of ANXA8, ANXA2, ANXA5 and ANXA6 in growing antler tip although this was not a comparative study of expression levels. In the AP cells, ANXA4 and ANXA5 are overexpressed 15-fold and two-fold, respectively, compared with FP cells, but the IPA analysis indicates MYC decreased expression of ANXA4 and ANXA5 [51], [61]. MYC could have the same effect on ANXA4 in PP cells. So the AP CELLS could be poised to differentiate down the chondrocyte pathway but are held in check and maintained in the proliferating state by MYC. ANXA2 could increase the expression of MYC [62] which would indicate an involvement in continued proliferation and the maintenance of stemness. The role of ANXA1, which is two-fold over-expressed is less clear. PI3K signalling is essential for translocation of ANXA1 to the cell membrane [63]. The 14-3-3 protein YWHAZ binds to ANXA1 [64] and it may mediate an anti-inflammatory effect by interacting with IL8 [65]. In the case of PP cells in these conditions the role of ANXA1 in the control of the cell cycle via the 14-3-3 pathways seems the most likely, implying that ANXA1 is involved in the proliferation of the cells.

Predictably, this study indicates a role for RA in antler regeneration. Extensive work has also shown the critical role of RA in amphibian limb regeneration [66]. Of the RA associated proteins upregulated by AP cells and PP cells, CRAPB2 binds to and is regulated by RA [67], [68], while the expression of CCT5, EEF1G, EEF1D, LAP3 and KRT1 is regulated by RA. EIF5A binding is regulated by RA. IPA shows a role for RA in AP cells and PP cells but not through Wnt signalling. Canonical Wnt signalling has a lesser role to play in the biology of the AP cells and PP cells. In the antler tip, some of the cells are differentiating into chondrocytes, while others are proliferating. This might explain the difference between the degree of Wnt signalling in the different stages. RA is involved in the upregulation of POU5F1 [69] which suggests it is involved in the maintenance of stemness in AP cells.

The IPA networks indicate that each of the extracellular proteins identified could interact with one of the transcription factors identified by Western blot. The transcription factors POU5F1 and NANOG are widely regarded as essential for stem cell maintenance have previously been identified in antler stem cells [2]. In the present study, we have confirmed their presence in both the cells and tissues and gained an insight into the extracellular molecules that could mediate or be mediated by their expression. In addition, we have confirmed the expression of SOX2, the third transcription factor associated with stem cell maintenance. The analysis also suggests that one way in which SOX2 could be maintaining the pluripotent is state and minimizing the effect of Wnt signalling by down regulating COL1A1.

Several of the pathways and proteins are significant in embryonic stem cell biology. The PI3K/Akt and ERK/MAPK pathways are important for maintaining self-renewal and pluripotency [70]–[71]. In contrast,POU5F1, SOX2 and NANOG act together to maintain stem cell pluripotency and self-renewal, while MYC appears to be essential for cell cycle progression [72]. The presence of these proteins in AP and PP cells and in the growing antler tip supports the notion that antler growth is a stem-cell mediated process and that antler stem cells share some properties of embryonic stem cells. In conclusion, we have identified a set of proteins unique to the antler stem cell niche using a shotgun approach. Further studies will demonstrate the relevance of these proteins in antler stem cell biology and antler regeneration. Furthermore, this work identifies proteins involved in the early stages of pedicle and antler growth and regeneration and assigns plausible connections within signalling pathways using IPA analysis.

## Supporting Information

Figure S1 **Pedicle periosteum (PP) and antlerogenic periosteum (AP).** A: PP deletion. PP was peeled off from a pedicle stump after being cut into strips (arrow). B: PP-less pedicle failed to give rise to a regenerating antler (arrow), although the control pedicle formed a two-tine antler. C: AP deletion. AP was peeled off from the future pedicle growth region prior to initiation of the pedicle (arrow). D: No pedicle and antler were formed from the AP-less future pedicle growth region, whereas a two-tine ectopic antler was formed from the AP-grafted-forehead-region (arrow).
(TIF)

Figure S2 **Merged networks of the APC proteome.** Note that most of the proteins identified could be linked together functionally and there could be crosstalk between the various canonical networks involved. Proteins detected in the proteomic study are shown in grey to red. The deeper the red, the greater the level of expression. Proteins indicated by IPA analysis and subsequently detected by western blots are shown in yellow. Proteins shown in white are indicated as involved in the network but have not yet been detected.
(TIF)

Figure S3 **Merged networks of the PPC proteome.** For detailed annotation of the figure, please refer to **Figure S2**.
(TIF)

Table S1 **Proteins expressed by AP cells indicating expression levels compared with FP cells.** Proteins are grouped by cell function or cell location. ON = present in APCs but not FPCs.
(DOCX)

Table S2 **Proteins expressed by PP cells indicating expression levels compared with FP cells.** Proteins are grouped by cell function or cell location. ON = present in PPCs but not FPCs.
(DOCX)
