# Supplementary figures and images for: Proteomes and Signalling Pathways of Antler Stem Cells
Source: PLoS One. 2012 Jan 18;7(1):e30026. doi: 10.1371/journal.pone.0030026 (PMC3261186; doi:10.1371/journal.pone.0030026)

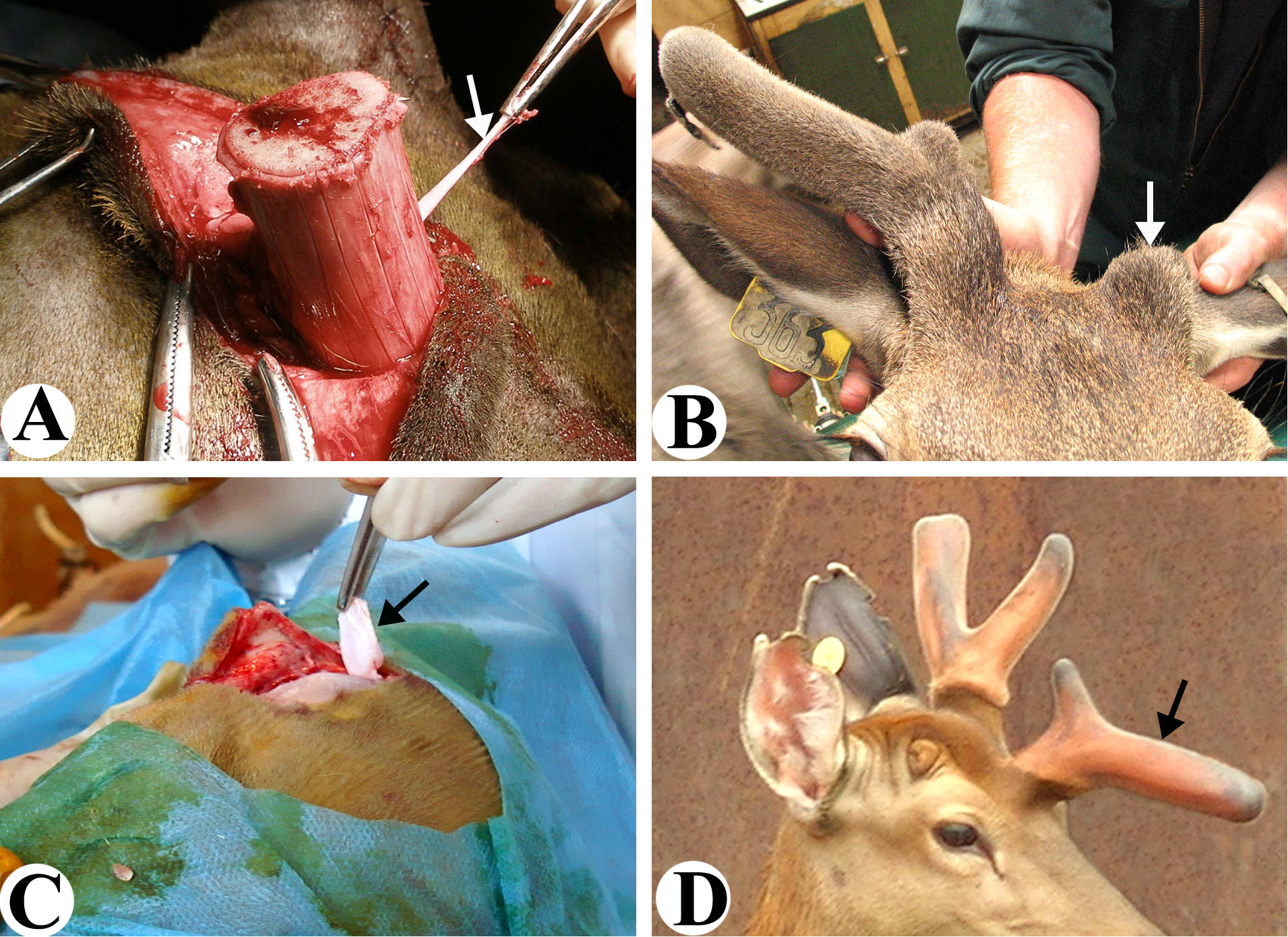

Supplement: Figure S1 — Pedicle periosteum (PP) and antlerogenic periosteum (AP). A: PP deletion. PP was peeled off from a pedicle stump after being cut into strips (arrow). B: PP-less pedicle failed to give rise to a regenerating antler (arrow), although the control pedicle formed a two-tine antler. C: AP deletion. AP was peeled off from the future pedicle growth region prior to initiation of the pedicle (arrow). D: No pedicle and antler were formed from the AP-less future pedicle growth region, whereas a two-tine ectopic antler was formed from the AP-grafted-forehead-region (arrow). (TIF) [file pone.0030026.s001.tif]

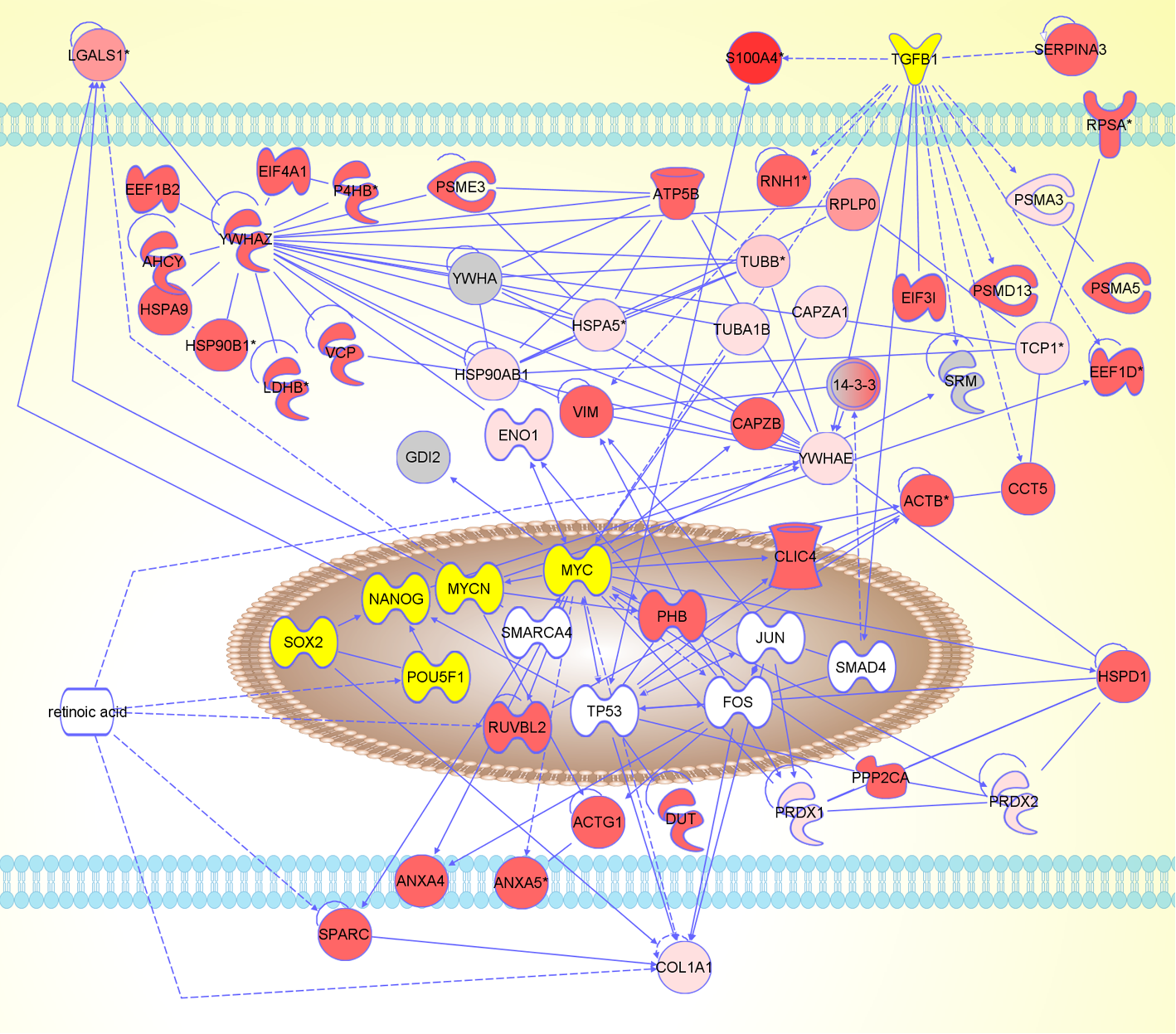

Supplement: Figure S2 — Merged networks of the APC proteome. Note that most of the proteins identified could be linked together functionally and there could be crosstalk between the various canonical networks involved. Proteins detected in the proteomic study are shown in grey to red. The deeper the red, the greater the level of expression. Proteins indicated by IPA analysis and subsequently detected by western blots are shown in yellow. Proteins shown in white are indicated as involved in the network but have not yet been detected. (TIF) [file pone.0030026.s002.tif]

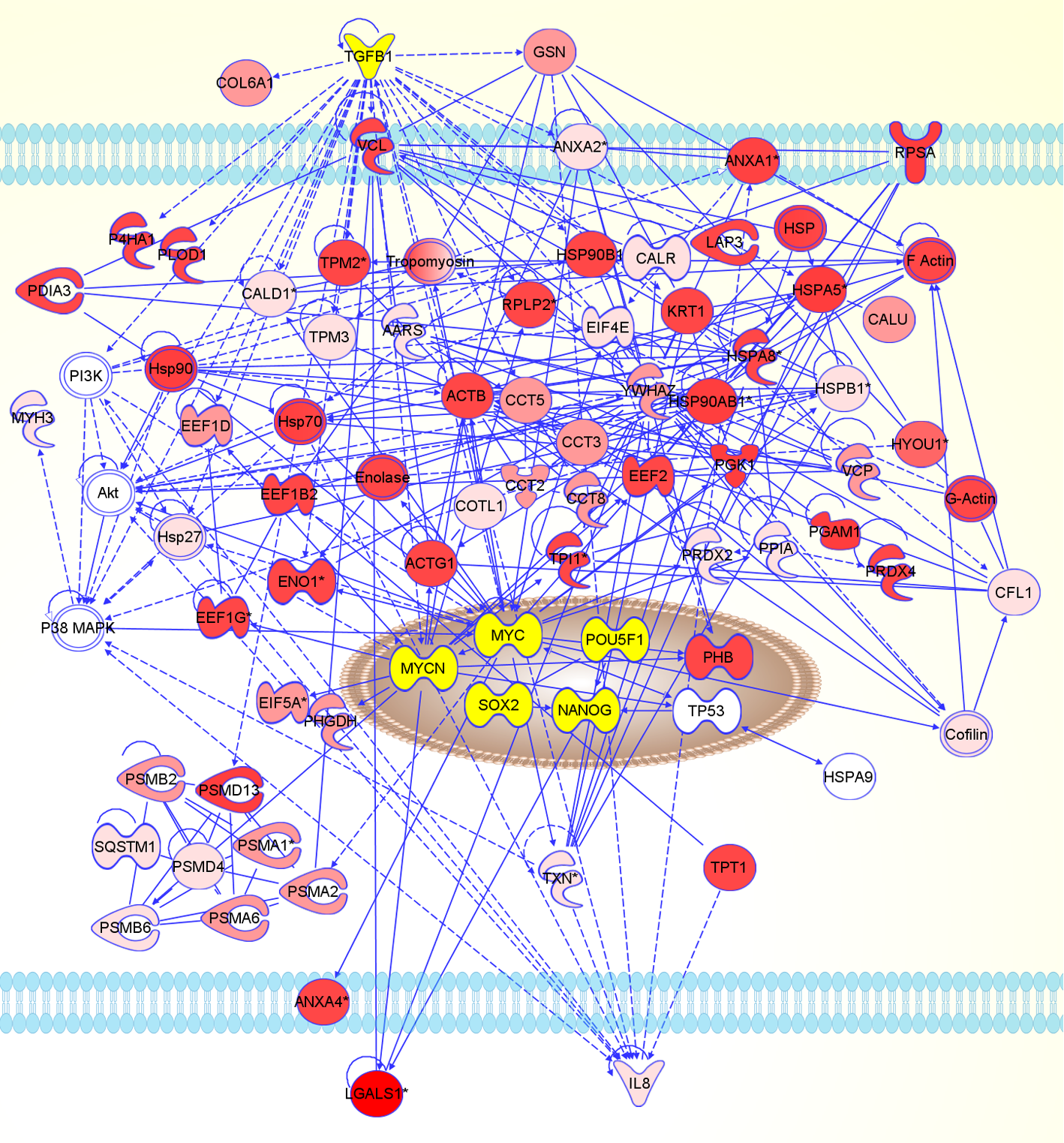

Supplement: Figure S3 — Merged networks of the PPC proteome. For detailed annotation of the figure, please refer to Figure S2. (TIF) [file pone.0030026.s003.tif]
