# Supplementary material for: Proteomes and Signalling Pathways of Antler Stem Cells
Source: PLoS One. 2012 Jan 18;7(1):e30026. doi: 10.1371/journal.pone.0030026 (PMC3261186; doi:10.1371/journal.pone.0030026)
Supplement: Table S1 — Proteins expressed by AP cells indicating expression levels compared with FP cells. Proteins are grouped by cell function or cell location. ON = present in APCs but not FPCs. (DOCX) [file pone.0030026.s004.docx]

**Table S1.** Proteins expressed by AP cells indicating expression levels compared with FP cells. Proteins are grouped by cell function or cell location. ON = present in APCs but not FPCs

| **Accession number** | **Protein name** | **Gene name (used in IPA analysis)** | **Function** | **Expression fold change versus FPCs** | **Matched/**  **searched** | **Sequence coverage** | **score** | **E value** |
| --- | --- | --- | --- | --- | --- | --- | --- | --- |
| **Cytoskeleton and cell motility** | | | | | | | | |
| gi\|4506765 | S100A4 | S100A4 | \| \| motility, apoptosis \| \| --- \| \| \| --- \| --- \| | ON | 7/34 | 39% | 93 | 0.0034 |
| gi\|1703 | Actin beta | ACTB | nucleotide binding; structural constituent of cytoskeleton; protein binding; | 2 | 7/46 | 46% | 76 | 0.016 |
| gi\|1703118 | actin gamma 1 | ACTG1 | cell motility; sarcomere organization | 5 | 15/46 | 42% | 154 | 3.8e-10 |
| gi\|4826659 | F-actin capping protein beta subunit | CAPZB | actin binding proteolysis; cell motility; actin cytoskeleton organization and biogenesis; barbed-end actin filament capping | ON | 10/38 | 47% | 109 | 8.9e-06 |
| gi\|18088719 | Tubulin, beta | TUBB | \| \| microtubule-based movement; mitotic spindle assembly \| \| --- \| \| \| --- \| --- \| | 2 | 16/83 | 40% | 144 | 3.8e-09 |
| gi\|549052 | Tubulin alpha chain | TUBA1B |  | 2 | 20/100 | 57% | 158 | 1.1e-09 |
| gi\|110347570 | Vimentin | VIM | \| \| structural constituent of cytoskeleton; protein binding \| \| --- \| \| \| --- \| --- \| | 15 | 27/100 | 54% | 164 | 2.7e-10 |
| gi\|114672411 | Myosin light chain isoform 2 | MRLC2 |  | ON | 7/53 | 49% | 75 | 0.2 |
| **Anti-apoptosis** | | | | | | | | |
| gi\|119938323 | Rho GDP dissociation inhibitor isoform 3 | ARHGDIA | cell motility; negative regulation of cell adhesion; Rho protein signal transduction | 2 | 8/41 | 34% | 97 | 0.00015 |
| gi\|28189813 | Translationally controlled tumor protein | TPT1 | \| \| calcium ion transport; \| \| --- \| \| \| --- \| --- \| | ON | 7/70 | 27% | 77 | 0.15 |
| gi\|73974186 | tyrosine 3/tryptophan 5 -monooxygenase activation protein, zeta polypeptide isoform 5 | YWHAZ | \| \| protein targeting; signal transduction \| \| --- \| \| \| --- \| --- \| | 15 | 14/79 | 54% | 98 | 0.00017 |
| gi\|55824576 | 14-3-3  tyrosine 3/tryptophan 5 -monooxygenase activation protein, theta polypeptide | YWHAQ | Protein targeting, signal transduction, apoptosis, cell spreading, differentiation. | 15 | 17/68 | 53% | 156 | 2.4e-10 |
| gi\|5803225 | tyrosine 3/tryptophan 5 -monooxygenase activation protein, epsilon polypeptide | YWHAE | intracellular signaling cascade | 15 | 15/51 | 57% | 121 | 1.4e-09 |
| gi\|115495027 | heat shock 70kDa protein 5 | HSPA5 | ER overload response; negative regulation of caspase activity | 2 | 27/87 | 43% | 208 | 1.5e-15 |
| **Protein folding** | | | | | | | | |
| gi\|27807263 | tumour rejection antigen (gp96) | HSP90B1 | \| \| response to hypoxia; anti-apoptosis; protein transport; sequestering of calcium ion \| \| --- \| \| \| --- \| --- \| | 2 | 34/61 | 40% | 281 | 5.6e-23 |
| gi\|27806501 | procollagen-proline, 2-oxoglutarate 4-dioxygenase | P4HB | protein disulfide isomerase activity; procollagen-proline 4-dioxygenase activity; | 2 | 33/71 | 55% | 315 | 2.2e-25 |
| gi\|71152402 | 60 kDa heat shock protein, mitochondrial precursor | HSPD1 | Nucleotide binding;regulation of apoptosis | 2 | 19/56 | 33% | 160 | 7.1e-11 |
| gi\|73970902 | 75 kDa glucose regulated protein | HSPA9 | nucleotide binding;anti-apoptosis | 15 | 21/43 | 41% | 189 | 8.9e-14 |
| gi\|51859516 | Heat shock 90kDa protein 1 | HSP90AB1 | nucleotide binding; nitric-oxide synthase regulator activity; TPR domain binding; positive regulation of nitric oxide biosynthetic process | 3 | 34/56 | 51% | 354 | 2.7e-29 |
| gi\|149743996 | T-complex protein 1, alpha subunit | TCP1 | protein folding; tubulin folding | 2 | 17/78 | 31% | 98 | 0.00017 |
| gi\|194676636 | chaperonin containing TCP1, subunit 5 | TCP1 | \| \| protein folding; tubulin folding \| \| --- \| \| \| --- \| --- \| | 2 | 20/65 | 31% | 128 | 1.1e-07 |
| **Translation** | | | | | | | | |
| gi\|2293577 | acidic ribosomal phosphoprotein PO | RPLP0 | RNA binding; structural constituent of ribosome; ribosome biogenesis and assembly | 2 | 13/79 | 47% | 106 | 1.8e-05 |
| gi\|73621050 | Eukaryotic initiation factor 4A-I | EIF4A1 | nucleotide binding ATP-dependent helicase activity; hydrolase activity | 2 | 22/63 | 57% | 187 | 1.4e-13 |
| gi\|57164211 | translational elongation factor 1 beta | EEF1B2 | Translation elongation. Binds TPT1 and YWHAT | 15 | 12/29 | 45% | 72 | 0.39 |
| gi\|57164211 | Elongation factor 1-delta | EEF1D | positive regulation of I-kappaB kinase/NF-kappaB cascade | 15 | 11/52 | 36% | 105 | 3e-05 |
| gi\|4503513 | eukaryotic translation initiation factor 3, subunit 2 beta, 36kDa | EIF3I |  | 15 | 11/51 | 39% | 104 | 0.00027 |
| **Metabolic and biosynthesis** | | | | | | | | |
| gi\|109095927 | lactate dehydrogenase B | LDHB | anaerobic glycolysis | 2 | 8/43 | 23% | 82 | 0.049 |
| gi\|66773956 | Peroxiredoxin-1 | PRDX1 | Redox regulation | 15 | 10/40 | 33% | 125 | 2.2e-07 |
| gi\|27807469 | peroxiredoxin 2 | PRDX2 | Redox regulation | 15 | 11/37 | 14% | 78 | 0.014 |
| gi\|28461221 | ATP synthase, H+ transporting, mitochondrial F1 complex | ATP5B | Generation of precursor metabolites | ON | 19/41 | 59% | 208 | 1.5e-15 |
| gi\|78042498 | phosphoglycerate dehydrogenase | PHGDH | L-serine biosynthetic process; brain development | 2 | 14/70 | 20% | 92 | 0.00059 |
| gi\|77735583 | S-adenosylhomocysteine hydrolase | AHCY | one-carbon compound metabolic process | 2 | 21/78 | 42% | 180 | 9.6e-13 |
| gi\|4139392 | Chain A, Cytochrome Bc1 Complex | UQCRC1 | ubiquinol-cytochrome-c reductase activity; oxidoreductase activity  electron transport; aerobic respiration | 15 | 23/62 | 53% | 73 | 0.038 |
| **Ubiquitin** | | | | | | | | |
| gi\|7106387 | Proteasome subunit alpha type 5 | PSMA5 | ubiquitin-dependent protein catabolic process | ON | 8/35 | 40% | 92 | 0.0005 |
| gi\|73965642 | proteasome (prosome, macropain) activator subunit 3 | PSME3 | proteasome activator activity | ON | 8/46 | 24% | 89 | 0.0012 |
| gi\|114653229 | proteasome alpha 3 subunit isoform 5 | PSMA3 | threonine endopeptidase activity; protein binding | ON | 11/60 | 40% | 118 | 1.5e-06 |
| gi\|3618343 | 26S proteasome subunit p40.5 | PSMD13 | meiosis I | ON | 13/66 | 34% | 130 | 9.6e-08 |
| **Cytoplasm** | | | | | | | | |
| gi\|74354056 | Enolase 1 | ENO1 | \| \| negative regulation of transcription from RNA polymerase II promoter; glycolysis; transcription; negative regulation of cell growth \| \| --- \| \| \| --- \| --- \| | 2 | 8/30 | 24% | 81 | 0.052 |
| gi\|84370035 | signal sequence receptor, delta | SSR4 | Intracellular protein transport | 2 | 6/30 | 38% | 86 | 0.019 |
| gi\|78369510 | ribonuclease/angiogenin inhibitor 1 [Mus musculus] | RNH1 | \| \| protein binding; ribonuclease inhibitor activity \| \| --- \| \| \| --- \| --- \| | 15 | 10/37 | 28% | 98 | 0.0011 |
| gi\|73971212 | valosin-containing protein isoform 4 | VCP | double-strand break repair; transport; caspase activation; regulation of apoptosis; | 2 | 32/100 | 43% | 267 | 1.4e-20 |
| gi\|73985921 | epsilon subunit of coatomer protein complex isoform c | COPE | retrograde Golgi to ER; transport | 15 | 9/53 | 28% | 99 | 0.00012 |
| gi\|122692293 | chloride intracellular channel 4 | CLIC4 | chloride transport; cell differentiation | ON | 11/55 | 47% | 116 | 2.4e-06 |
| gi\|134024617 | reticulocalbin 1 precursor | RCN1 | calcium ion binding | ON | 8/43 | 27% | 79 | 0.011 |
| gi\|78369510 | ribonuclease/angiogenin inhibitor | RNH1 | ribonuclease inhibitor activity regulation of angiogenesis | 15 | 10/37 | 28% | 110 | 9.6e-06 |
| gi\|61554531 | protein phosphatase 2, catalytic subunit, alpha isoform | PPP2CA | regulation of progression through cell cycle | 15 | 13/40 | 46% | 68 | 0.1 |
| gi\|78369496 | dendritic cell protein | EIF3M | herpesvirus entry mediator | 15 | 7/41 | 12% | 74 | 0.035 |
| gi\|27806353 | tryptophanyl-tRNA synthetase | WARS | negative regulation of cell proliferation | 15 | 10/56 | 20% | 88 | 0.0014 |
| gi\|1363133 | GDP dissociation inhibitor 2 | GDI2 | small GTPase mediated signal transduction | 2 | 7/33 | 53% | 81 | 0.055 |
| **Plasma membrane** | | | | | | | | |
| gi\|120474983 | annexin A5 | ANAXA5 | anti-apoptosis; signal transduction | 2 | 17/76 | 52% | 176 | 2.4e-12 |
| gi\|260138 | annexin V CaBP37 isoform | ANAXA5 | anti-apoptosis; signal transduction; | 2 | 12/46 | 34% | 119 | 8.6e-06 |
| gi\|14278334 | Annexin IV | ANXA4 | anti-apoptosis; signal transduction; | 15 | 34/58 | 72% | 122 | 6.1e-07 |
| gi\|119584991 | ribosomal protein SA, isoform CRA_c | RPSA | \| \| translation; cell adhesion; cell surface receptor linked signal transduction \| \| --- \| \| \| --- \| --- \| | 6/42 | 11/55 | 66% | 78 | 0.011 |
| **Nucleus** | | | | | | | | |
| gi\|62751970 | chloride intracellular channel 1 | CLIC1 | voltage-gated chloride channel activity; chloride transport; signal transduction | 15 | 14/60 | 50% | 164 | 2.8e-11 |
| gi\|4505773 | prohibitin | PHB | negative regulation of cell proliferation | ON | 14/77 | 65% | 132 | 6.1e-08 |
| gi\|118150864 | dUTP pyrophosphatase | DUT | magnesium ion binding; DNA replication | 15 | 5/50 | 35% | 72 | 0.067 |
| gi\|73948016 | 48-kDa TATA box-binding protein-interacting protein | RUVBL2 | DNA repair; DNA recombination; chromatin modification | 15 | 34/64 | 59% | 121 | 5.6e-07 |
| **Extra cellular space** | | | | | | | | |
| gi\|47779226 | Galectin-1 | LGALS1 | \| \| signal transducer activity; protein binding; sugar binding \| \| --- \| \| \| --- \| --- \| | 15 | 8/48 | 50% | 127 | 1.4e-06 |
| gi\|27806941 | serine (or cysteine) proteinase inhibitor, clade A | SERPINA1 | Extra-cellular protease inhibition   \| \| chemotaxis, proliferation, migration \| \| --- \| \| \| --- \| --- \| | 2 | 16/59 | 39% | 204 | 2.7e-14 |
| gi\|31340900 | serpin peptidase inhibitor, clade A | SERPINA3 | \| \| deposition, disaggregation, formation, chemotaxis, cell spreading \| \| --- \| \| \| --- \| --- \| | 2 | 16/59 | 39% | 204 | 2.8e-15 |
| gi\|77404252 | collagen, type I,  alpha 1 | COL1A1 | extracellular matrix structural constituent;; structural constituent of bone, epidermis development | ON | 24/52 | 25% | 169 | 8.9e-12 |
| gi\|73954161 | SPARC precursor | SPARC | \| \| ossification; transmembrane receptor protein tyrosine kinase signalling pathway \| \| --- \| \| \| --- \| --- \| | ON | 9/51 | 30% | 61 | 0.51 |
| **Unknown** | | | | | | | | |
| gi\|5453597 | F-actin capping protein alpha-1 subunit | CAPZA1 | Cell mobility, merging, internalization | 15 | 10/38 | 47% | 109 | 8.9e-06 |
| gi\|108997020 | spermidine synthase | SRM | spermidine synthase activity transferase activity | 15 | 8/63 | 29% | 80 | 0.0076 |
