# Supplementary material for: Proteomes and Signalling Pathways of Antler Stem Cells
Source: PLoS One. 2012 Jan 18;7(1):e30026. doi: 10.1371/journal.pone.0030026 (PMC3261186; doi:10.1371/journal.pone.0030026)
Supplement: Table S2 — Proteins expressed by PP cells indicating expression levels compared with FP cells. Proteins are grouped by cell function or cell location. ON = present in PPCs but not FPCs. (DOCX) [file pone.0030026.s005.docx]

**Table S2.** Proteins expressed by PP cells indicating expression levels compared with FP cells. Proteins are grouped by cell function or cell location. ON = present in PPCs but not FPCs

| **GI #** | **name** | **Gene name** |  | **Fold**  **change** | **Matched/**  **searched** | **Sequence coverage** | **score** | **E value** |
| --- | --- | --- | --- | --- | --- | --- | --- | --- |
| **Cell motility** | | | | | | | | |
| gi\|109492380 | Actin, Cytoplasmic 2 | ACTG1 | sarcomere organization | 2 | 11/46 | 24% | 92 | 0.00047 |
| gi\|109914418 | Actin, cytoplasmic, beta 1 | ACTB | Protein and ATP binding | 2 | 10/49 | 43% | 98 | 0.00014 |
| gi\|115432192 | Alanyl-tRNA synthetase | AARS | alanine-tRNA ligase activity; tRNA processing, binds YWHAZ | 2 | 13/35 | 17% | 92 | 0.0005 |
| gi\|113594 | Aldose Reductase | AKR1B1 | aldehyde reductase activity; carbohydrate metabolic process | ON | 13/32 | 46% | 150 | 6.9e-09 |
| gi\|77539778 | Alpha Actinin 4 | ACTN4 | integrin binding; calcium ion binding, regulation of progression through cell cycle; regulation of apoptosis; positive regulation of pinocytosis; actin filament bundle formation; regulation of cell motility | 10 | 16/41 | 20% | 113 | 4.8e-06 |
| gi\|83778524 | beta-tropomyosin | TPM2 | actin binding; structural constituent of muscle,cytoskeleton; muscle thin filament tropomyosin | 2 | 13/28 | 32% | 131 | 5.6e-08 |
| gi\|27806279 | Caldesmon 1 | CALD1 | actin binding; calmodulin binding; tropomyosin binding; myosin binding, cell motility; muscle contraction | 2 | 14/44 | 25% | 106 | 1.8e-05 |
| gi\|77735435 | Chaperon containing TCP1, subunit 2, beta | TCP1 | tubulin folding | 10 | 16/57 | 40% | 120 | 7.1e-07 |
| gi\|114051526 | Coactosin-like 1 | COTL1 | actin binding; enzyme binding | ON | 8/37 | 56% | 92 | 0.00049 |
| gi\|73969816 | ARP2 actin-related protein 2 homolog | ACTR2 | structural molecule activity, cell motility | 10 | 6/31 | 27% | 74 | 0.028 |
| gi\|114624373 | Tropomysin 2 | TPM2 | Actin binding, protein binding, structural constituent of muscle | 10 | 10/30 | 23% | 81 | 0.006 |
| gi\|73961067 | Tropomyosin 3 | TPM3 | cell motility; regulation of muscle contraction | 10 | 15/28 | 60% | 208 | 1.1e-15 |
| gi\|109096498 | Tubulin Alpha, 1c | TUBA1C | microtubule-based movement; protein polymerization | 2 | 10/38 | 25% | 72 | 0.046 |
| gi\|2119276 | Tubulin Beta | TUBB | cell motility; microtubule-based movement; | 2 | 11/39 | 32% | 102 | 4.5e-05 |
| gi\|14389299 | vimentin | VIM | structural constituent of cytoskeleton; protein binding cell motility | 2 | 24/75 | 54% | 178 | 1.1e-12 |
| **Translation** | | | | | | | | |
| gi\|33383425 | Eukaryotic initiation factor 5A, isoform 1 | EIF5A | translation initiation factor activity | 10 | 6/38 | 25% | 71 | 0.045 |
| gi\|4503483 | Eukaryotic translation Iniation Factor 2 | EEF2 | nucleotide binding; translation elongation factor activity; | 2 | 12/37 | 16% | 88 | 0.01 |
| gi\|62460568 | Eukaryotic Translation Initiation Factor 1, beta 2 | EEF1B2 | translation elongation factor activity | 2 | 7/35 | 45% | 81 | 0.0073 |
| gi\|95769122 | Eukaryotic Translation Initiation Factor 1, gamma | EEF1G | translation elongation factor activity | 2 | 13/40 | 29% | 114 | 3.8e-06 |
| gi\|119626485 | Eukaryotic Translation Initiation Factor 4E | EIF4E | RNA cap binding; translation initiation factor activity | ON | 8/33 | 31% | 88 | 0.0011 |
| gi\|148680528 | Eukaryotic translation initiation factor 5A | EIF5A | translation initiation factor activity | ON | 6/28 | 48% | 91 | 0.00056 |
| gi\|27807407 | ferritin, light polypeptide | FTL | ferric iron binding; iron ion transport and homeostasis | ON | 5/19 | 40% | 73 | 0.045 |
| gi\|29135329 | Glutathione S-transferase pi | GSTP1 | anti-apoptosis; central nervous system development; | 10 | 7/41 | 43% | 71 | 0.0085 |
| gi\|147902079 | Glycyl-tRNA synthetase | GARS | glycine-tRNA ligase activity; | 1 | 11/36 | 17% | 72 | 0.054 |
| gi\|27807523 | ribosomal protein P2 | RPLP2 | translational elongation, large ribosomal subunit | 2 | 6/35 | 79% | 81 | 0.0057 |
| **Protein folding** | | | | | | | | |
| gi\|115495027 | Heat Shock 70kDa Protein 5 (GRP78) | HSPA5 | anti-apoptosis; ER overload response; negative regulation of caspase activity | 1 | 21/52 | 36% | 188 | 1.1e-13 |
| gi\|123647 | Heat Shock cognate 71kDa Protein 8 | HSPA8 | regulation of progression through cell cycle; chaperone cofactor-dependent protein folding | 5 | 10/42 | 21% | 79 | 0.0087 |
| gi\|40556608 | Heat Shock Protein 1, beta | HSP90AB1 | endoplasmic reticulum stress response, migration, apoptosis, angiogenesis, proliferation | 2 | 17/55 | 25% | 120 | 7.1e-07 |
| gi\|85542053 | Heat Shock protein beta-1 (HspB1) | HSPB1 | apoptosis, migration, blebbing, cell movement, reorganization | 2 | 8/23 | 48% | 113 | 3.4e-05 |
| gi\|12654329 | HSP90AA1 protein | HSP90AA1 | differentiation, endoplasmic reticulum stress response, migration, transformation, shape change, angiogenesis | 10 | 26/54 | 27% | 153 | 3.5e-10 |
| gi\|50979166 | heat shock protein 90kDa beta (Grp94), member 1 | HSP90B1 | response to hypoxia; protein folding; anti-apoptosis; protein transport; sequestering of calcium ion | 2 | 15/51 | 32% | 138 | 1.1e-08 |
| gi\|119907132 | Hyou1 Protein | HYOU1 | response to stress, apoptosis | 2 | 13/30 | 18% | 92 | 0.00049 |
| gi\|114051908 | FK506 Binding Protein 10, 65kDa | FKBP10 | peptidyl-prolyl cis-trans isomerase activity | 2 | 10/80 | 23% | 94 | 0.00027 |
| gi\|109940321 | chaperonin containing TCP1, subunit 3 (gamma) | CCT3 | ATP binding, unfolded protein binding | 10 | 9/36 | 17% | 72 | 0.059 |
| gi\|194676636 | chaperonin containing TCP1, subunit 5 (epsilon) | CCT5 | ATP binding, unfolded protein binding, regulated by retinoic acid | ON | 13/39 | 28% | 99 | 9.3e-05 |
| **Nucleus** | | | | | | | | |
| gi\|73983054 | Cofilin-1 isoform 2 | CFL1 | actin binding; anti-apoptosis; Rho protein signal transduction. | ON | 6/30 | 52% | 83 | 0.0036 |
| gi\|62751970 | Chloride Intracellular channel 1 | CLIC1 | ion transport; chloride transport; signal transduction | 2 | 11/44 | 46% | 123 | 3.5e-07 |
| \| gi\|10911422 \| \| --- \| | Prohibitin isoform 3 | PHB | transcriptional repressor activity  signal transduction; negative regulation of cell proliferation, histone deacetylation; regulation of apoptosis | 2 | 12/41 | 60% | 155 | 2.2e-10 |
| **Protein ubiquitination pathway** | | | | | | | | |
| gi\|13786158 | Proteasome (prosome,macropain) 26S subunit, non-ATPase, 4 | PSMD4 | proteasome complex | ON | 7/28 | 24% | 66 | 0.16 |
| gi\|73982052 | 26S Proteosome non-Atpase regulatory subunit 13 | PSMD13 | protein binding, meiosis I | 2 | 10/37 | 30% | 90 | 0.00074 |
| gi\|15663105 | proteasome 26S non-ATPase subunit 8 | PSMD8 | proteasome complex | ON | 8/33 | 22% | 75 | 0.032 |
| gi\|4506179 | Proteasome alpha 1, isoform 2 | PSAT1 | amino acid biosynthetic process | 10 | 9/33 | 30% | 95 | 0.00021 |
| gi\|4506181 | Proteasome alpha 2 subunit | PSMA2 | ubiquitin-dependent protein catabolic process, proteasome core complex | 10 | 7/25 | 41% | 93 | 0.00038 |
| gi\|109083329 | Proteasome subunit alpha,type 6 | PSMA6 | As above | 10 | 5/22 | 31% | 72 | 0.057 |
| gi\|62751339 | Proteasome subunit beta, type 2 | PSMB2 | As above | 10 | 8/22 | 26% | 92 | 0.0046 |
| gi\|77735687 | Proteosome subunit beta | PSMB6 | As above | ON | 5/29 | 24% | 68 | 0.053 |
| **Cytoplasm** | | | | | | | | |
| gi\|230115 | Leucine Aminopeptidase chain A | LAP3 | proteolysis; protein metabolic process | 2 | 7/24 | 14% | 77 | 0.013 |
| gi\|73980351 | Mitochondrial inner membrane protein (Mitofilin) isoform 9 | IMMT | Regulates calmodulin | 10 | 8/36 | 15% | 98 | 0.00011 |
| gi\|109113275 | Myosin Heavy Chain, Fast skeletal muscle, embryonic isoform 1 | MYH3 | muscle contraction; muscle development; actin filament-based movement | ON | 14/32 | 9% | 77 | 0.013 |
| gi\|52695491 | Oxidized Bovine Microsomal Cytochrome B5 Mutant V45h chain A | CYB5A | heme binding, electron transport; transport | 1 | 4/22 | 67% | 73 | 0.037 |
| gi\|2780749 | peroxiredoxin 2 | PRDX2 | Redox regulation, response to oxidative stress; regulation of apoptosis | ON | 11/37 | 14% | 78 | 0.014 |
| gi\|27806085 | Peroxiredoxin 4 | PRDX4 | thioredoxin peroxidase activity; peroxiredoxin activity | 2 | 9/41 | 27% | 85 | 0.0022 |
| gi\|52783777 | Phosphoglycerate Kinase 1 | PGK1 | glycolysis; phosphorylation | 2 | 10/35 | 30% | 95 | 0.00022 |
| gi\|11957032 | Phosphoglycerate mutase 1 (brain), isoform CRA_a | PGAM1 | glycolysis; metabolic process | 2 | 6/23 | 29% | 66 | 0.02 |
| gi\|56710325 | Cellular retinoic acid binding protein 2 | CRABP2 | retinoic acid binding; signal transduction; epidermis development; embryonic forelimb morphogenesis; retinoic acid metabolic process | ON | 9/33 | 55% | 111 | 5.6e-06 |
| gi\|75812922 | Chaperonin containing TCP1, subunit 8, theta | CCT2 | regulation of progression through cell cycle | 10 | 15/36 | 28% | 148 | 1.1e-09 |
| gi\|8515718 | Calumenin | CALU | calcium ion binding | 10 | 11/39 | 41% | 112 | 4.5e-06 |
| gi\|7767529 | Cyclophilin I | PPIA | protein folding, genome replication | ON |  |  |  |  |
| gi\|71153760 | D-3-phosphoglycerate dehydrogenase (3-PGDH | PHGDH | brain development; amino acid biosynthetic process | 10 | 8/32 | 27% | 82 | 0.049 |
| gi\|109481384 | Coatomer subunit zeta-1 | COPZ1 | intracellular protein transport | 1 | 17/52 | 51% | 171 | 5.6e-12 |
| gi\|729433 | Protein Disulfide Isomerase | PDIA3 | protein retention in ER; signal transduction; positive regulation of apoptosis; cell redox homeostasis | 2 | 14/52 | 26% | 82 | 0.0068 |
| gi\|28603774 | Rho GDP dissocaiation inhibitor, alpha | ARHGDIA | anti-apoptosis; cell motility; negative regulation of cell adhesion; Rho protein signal transduction | ON | 10/37 | 52% | 111 | 5.6e-06 |
| gi\|27807523 | ribosomal protein P2 | RPLP2 | translational elongation, large ribosomal subunit | 2 | 6/35 | 79% | 81 | 0.0057 |
| gi\|119584991 | Ribosomal Protein SA | RPSA | structural constituent of ribosome | 2 | 12/54 | 77% | 180 | 7.1e-13 |
| gi\|110626169 | sequestosome 1 | SQSTM1 | ubiquitin-dependent protein catabolic process | ON | 6/28 | 28% | 64 | 0.032 |
| gi\|76655873 | Rab coupling protein | RAB11FIP1 | regulate Rab GTPases | ON | 8/15 | 12% | 95 | 0.00023 |
| gi\|119915487 | superkiller viralicidic activity 2-like | SKIV2L | Purine metabolism, | ON | 6/25 | 9% | 76 | 0.016 |
| gi\|73970894 | heat shock 70kDa protein 9 (mortalin) | HSPA9 | control of cell proliferation, apoptosis, colony formation, differentiation | 2 | 15/56 | 33% | 101 | 5.6e-05 |
| gi\|47523692 | Thioredoxin | TXN | electron transport; cell motility; signal transduction; cell-cell signaling; cell proliferation; cell redox homeostasis | ON | 7/45 | 45% | 88 | 0.0014 |
| gi\|84579841 | TNF receptor-associated protein 1 | TRAP1 | tumor necrosis factor receptor binding; apoptosis | 2 | 11/29 | 16% | 76 | 0.16 |
| gi\|73954721 | Transgelin | TAGLN | actin binding; muscle development | ON | 10/41 | 39% | 107 | 1.4e-05 |
| gi\|57164211 | eukaryotic translation elongation factor 1 delta | EEF1D | signal transducer activity, translational elongation | 10 | 7/30 | 29% | 77 | 0.021 |
| gi\|28189813 | tumor protein, translationally-controlled 1 | TPT1 | proliferation, quantity, activation, recruitment, migration, cell death, growth, apoptosis | ON | 6/32 | 29% | 75 | 0.024 |
| gi\|61888856 | triosephosphate isomerase 1 | TPI1 | fatty acid biosynthetic process; metabolic process | 2 | 14/41 | 69% | 182 | 6.1e-13 |
| gi\|73974186 | tyrosine 3-monooxygenase/tryptophan 5-monooxygenase activation protein, zeta polypeptide | YWHAZ | transcription factor binding, protein targeting; anti-apoptosis; signal transduction | 10 | 15/38 | 46% | 155 | 2.2e-10 |
| gi\|115432192 | Alanyl-tRNA synthetase | AARS | alanine-tRNA ligase activity; tRNA processing, binds YWHAZ | ON | 13/35 | 17% | 92 | 0.0005 |
| gi\|77735541 | valosin-containing protein | VCP | protein ubiquitination; ER-associated protein catabolic process; unfolded protein response; regulation of apoptosis; establishment of protein localization | 10 | 14/54 | 23% | 79 | 0.0087 |
| gi\|27807407 | ferritin, light polypeptide | FTL | ferric iron binding; iron ion transport and homeostasis | ON | 13/48 | 30% | 94 | 0.00043 |
| gi\|29135329 | Glutathione S-transferase pi | GSTP1 | anti-apoptosis; central nervous system development; | 10 | 7/41 | 43% | 79 | 0.0085 |
| gi\|147902079 | Glycyl-tRNA synthetase | GARS | glycine-tRNA ligase activity; | 2 | 11/39 | 17% | 72 | 0.054 |
| **Plasma membrane** | | | | | | | | |
| gi\|28189773 | 40S Ribosomal protein SA (p40 | RPSA | structural constituent of ribosome; laminin receptor activity, cell adhesion; cell surface receptor linked signal transduction | 2 | 7/33 | 37% | 94 | 0.003 |
| gi\|122144928 | Annexin A2 | ANAX2 | phospholipase inhibitor activity calcium-dependent phospholipid binding; cytoskeletal protein binding | ON | 6/41 | 51% | 171 | 5.6e-12 |
| gi\|4757756 | Annexin A2 isoform 2 | ANAX2 |  | ON | 17/52 | 51% | 171 | 5.6e-12 |
| gi\|48374083 | Annexin A4 | ANXA4 | calcium-dependent phospholipid binding, anti-apoptosis; signal transduction; negative regulation of coagulation | 2 | 19/42 | 54% | 205 | 2.2e-15 |
| gi\|74 | Annexin I | ANAX1 | phospholipase inhibitor activity, anti-apoptosis; cell motility; keratinocyte differentiation; regulation of cell proliferation; arachidonic acid secretion | 2 | 14/38 | 48% | 130 | 6.9e-07 |
| gi\|260138 | Annexin A5 | ANAX5 | anti-apoptosis; signal transduction; blood coagulation; negative regulation of coagulation | 2 | 16/57 | 45% | 150 | 7.1e-10 |
| **Extra cellular** | | | | | | | | |
| gi\|77404252 | Collagen, type 1, alpha | COL1A1 | extracellular matrix structural constituent; protein binding; structural constituent of bone | ON | 14/28 | 15% | 100 | 0.0001 |
| gi\|119887130 | Collagen, type VI, alpha 1 isoform 1 | COL6A1 | extracellular matrix structural constituent; cell adhesion, aggregation | 10 | 11/22 | 12% | 91 | 0.00054 |
| gi\|57164313 | Galectin-1 | LGALS1 | signal transducer activity; protein binding; sugar binding, regulation of apoptosis | 20 | 9/49 | 50% | 133 | 3.5e-08 |
| gi\|1352456 | Interleukin-8 precursor (IL-8) (CXCL8) (Neutrophil attractant protein 1) (NAP | IL8 | angiogenesis; cell motility;negative regulation of cell proliferation; regulation of cell adhesion; neutrophil chemotaxis and activation, | ON | 6/58 | 59% | 69 | 0.012 |
| gi\|114626487 | gelsolin isoform 1 | GSN | actin filament polymerization; actin filament severing; barbed-end actin filament capping | 10 | 10/28 | 16% | 75 | 0.022 |
| **Unknown** | | | | | | | | |
| gi\|27806723 | Calreticulin  ER and extracellular space | CALR | regulation of transcription; protein export from nucleus; cortical actin cytoskeleton organization and biogenesis; regulation of meiosis; regulation of apoptosis | ON | 12/35 | 30% | 134 | 2.7e-07 |
| gi\|122889667 | alanyl-tRNA synthetase domain containing 1 | AARSD1 |  | ON | 13/35 | 17% | 92 | 0.0005 |
